# Match-Fixing Causing Harm to Athletes on a COVID-19-Influenced Gambling Market: A Call for Research During the Pandemic and Beyond

**DOI:** 10.3389/fpsyg.2021.712300

**Published:** 2021-09-21

**Authors:** A Håkansson, C Jönsson, G Kenttä

**Affiliations:** ^1^Faculty of Medicine, Dept of Clinical Sciences Lund, Psychiatry, Lund University, Lund, Sweden; ^2^Region Skåne, Malmö Addiction Center, Clinical Sports and Mental Health Unit, Malmö, Sweden; ^3^FIFPRO (Global Representative for Professional Football Players), Hoofddorp, Netherlands; ^4^Spelarföreningen, National Representative for Football Players, Stockholm, Sweden; ^5^The Swedish School of Sport and Health Sciences, Stockholm, Sweden; ^6^School of Human Kinetics, University of Ottawa, Ottawa, ON, Canada; ^7^Swedish Sport Federation, Stockholm, Sweden

**Keywords:** gambling, problem gambling, COVID-19, match-fixing, crime, behavioral addiction, athletes mental health, sport psychology

## Abstract

Match-fixing, although not a new problem, has received growing attention during the COVID-19 pandemic, which has been reported in the media to have increased the risk of match-fixing events. Gambling is a well-documented addictive behavior, and gambling-related fraud, match-fixing, is a challenge to the world of sports. Most research on match-fixing has a judicial or institutional perspective, and few studies focus on its individual consequences. Nevertheless, athletes may be at particular risk of mental health consequences from the exposure to or involvement in match-fixing. The COVID-19 crisis puts a spotlight on match-fixing, as the world of competitive sports shut down or changed substantially due to pandemic-related restrictions. We call for research addressing individual mental health and psycho-social correlates of match-fixing, and their integration into research addressing problem gambling, related to the pandemic and beyond.

## Introduction

The COVID-19 pandemic, alongside its physical manifestations and its dramatic impact on global public health, has the potential to vastly affect mental health in many people world-wide (Holmes et al., 2020). Among many potential behavioral and mental health consequences, gambling behaviors have been highlighted as potentially changing during the pandemic (Håkansson et al., 2020). Gambling disorder is a well-established public health concern with extensive social and mental health consequences (Calado and Griffiths, 2016; Reith et al., 2019).

Likewise, the COVID-19 crisis has had an obvious impact on sports and leisure activities world-wide, including mental health consequences for young and adult athletes. In athletes, a major part of everyday life and career expectancies have been altered or cancelled. A major impact on the mental well-being of athletes therefore has been suspected (Haan et al., 2021).

## COVID-19, Gambling, Sports, and the Risk of Match-Fixing

COVID-19 affects the world of sports dramatically, including cancellations of events, or rigorous test protocols and restrictions, and many large-scale events take place in front of empty arenas (Wunderlich et al., 2021). Media have highlighted a possible surge in match-fixing issues during the pandemic, i.e., an increase of betting events where athletes are offered or threatened to engage in the fixing of a game, driven by gambling-related financial incentives. As high-level sports events were canceled early during the pandemic, even friendly games were highlighted by international gambling operators. Thus, in settings where some sports events still occurred, this led to a surprising attention to local low-tier soccer events, which are otherwise rarely associated with gambling industry (Associated Press, 2020; CBS Sports., 2020). In addition, the financial challenges experienced by teams and athletes due to COVID-19 are believed to make athletes and entire clubs more vulnerable to the risk of match-fixing, as stated by the major international monitoring service of Sportradar. Also, organized match-fixing may spread beyond larger team sports, and target individual sports, such as tennis (Independent Online, 2021) or e-sports (The Guardian., 2021). Altogether, the risk of undetected fraud may increase in lowly leagues during COVID-19, and the pandemic may direct match-fixing attempts toward sports typically not receiving attention by the media. Thereby, beyond the risk of match-fixing exposure for high-profile elite athletes, this may put young and amateur athletes into unfamiliar situations, leading to a risk of psychological impact for athletes on all levels. Hitherto, there is a lack of scientific data to support the impression of an increasing match-fixing problem during COVID-19, and this trend instead has been reported mainly as observations cited in a number of media reports (Associated Press, 2020; CBS Sports., 2020; South China Morning Post, 2020; Independent Online, 2021; The Guardian., 2021).

Match-fixing is not a new problem in sports (Huggins, 2018). Gambling patterns in recent years may reinforce risks of match-fixing; online and in-play sports betting are common, and modern match-fixing-related bets can be related to losing a full game or one specific part of a game, or could be associated with a team winning a game but with a smaller margin (Moriconi and de Cima, 2020). As in a movie based on a true story with Tim Donaghy, an experienced NBA referee arrested by the FBI, a blurred line between legal and illegal gambling adds to the difficulty of detecting and preventing match-fixing.

## Match-Fixing Affecting Mental Health in Athletes, Beyond Societal Consequences

Match-fixing has mainly received attention from a judicial or institutional perspective (Caneppele et al., 2020). In contrast, match-fixing has been sparsely addressed in research that examines problem gambling and its psychological aspects at the individual level. However, individual characteristics likely increase the vulnerability to match-fixing exposure; a lower income, being in a role where an individual’s action in a game may have limited visibility, and being on an amateur level where prevention and support services may be harder to access. Also, athletes may be at risk of mental distress because of mistakes made during the game, and where uncertainty may arise about whether these were voluntary or unvoluntary.

Recently, reports on individuals’ involvement in match-fixing have emerged (Moriconi and de Cima, 2020), and there is need to understand match-fixing in association with individual mental health. There is an obvious association between the world of sports, gambling markets, and gambling advertising, and this calls for an enhanced focus on gambling attitudes and prevention within the community of athletes (Vinberg et al., 2021). Moreover, scholars reported an association between having engaged in match-fixing and more accepting social norms toward match-fixing (Barboukis et al., 2020). Other researchers have argued that the risk of engaging in match-fixing may be particularly high in athletes with an own history of extensive gambling practices (O’Shea et al., 2021). Importantly, gambling problems have been shown to be over-represented in athletes, particularly in male athletes (Grall-Bronnec et al., 2016; Håkansson et al., 2018; Vinberg et al., 2020). Altogether, the gambling attitudes within and around the world of sports may elevate the risk of athletes actually engaging in this type of fraud. Thus, researchers have called for preventive interventions to include interventions regarding the gambling attitudes and practices of athletes themselves, and interventions against gambling problems in case they occur in athletes (O’Shea et al., 2021).

Mental health in elite athletes can be challenging, due to high expectations, stigma, and a high threshold for help-seeking (Reardon et al., 2019). There is also reason to suspect that mental health challenges in athletes world-wide may have increased further during the pandemic (Haan et al., 2021). Betting is common among soccer players and may include betting on own games. Although hitherto sparsely addressed, athletes with an own gambling problem theoretically could be at a particular risk of being exposed to match-fixing fraud (Moriconi and de Cima, 2020). In addition, during the COVID-19 crisis, match-fixing may put athletes at risk of further distress; unfortunately, current advice to athletes with respect do their mental well-being during the COVID-19 crisis fail to address match-fixing (AASP, 2020).

## Discussion

Given the likely mental impact of match-fixing on individual athletes, there is a need to address match-fixing in the prevention and assessment of mental health risk factors of athletes. Thus, there is reason to give greater attention to match-fixing both related to the increased risk highlighted during the COVID-19 crisis, as well as in more prolonged challenging financial situations beyond the pandemic. We call for research focusing on the individual psychological risk factors and consequences of match-fixing, such as studies on how individual at-risk gambling behavior among athletes may increase the risk of engaging in match-fixing. Risks and consequences of match-fixing should be addressed by professions and stakeholders working with sports psychology, both in relation to the COVID-19 and beyond. As part of this, it is of great importance to the world of sports and gambling to obtain structured scientifically credible data on the longitudinal development of match-fixing events, and whether a true increase was seen during the COVID-19 or not. Also, prevention and treatment of problem gambling in athletes likely benefit from a contextual knowledge and understanding of athletes’ everyday lives. In addition, there is need for strengthened routines and screening protocols for the early detection and intervention in athletes at risk of match-fixing involvement, as well as advice about athletes’ own risk of problem gambling. The latter may involve programs similar to anti-doping programs, in this case addressing attitudes to gambling within sports, such as athletes’ betting on their own games (Vinberg et al., 2020, 2021). Alert tools facilitating the prevention of match-fixing have been suggested and may include “whistle blower” systems (Barkoukis et al., 2019), making it possible to reveal ongoing or attempted match-fixing events. Such a “whistle blower” function should likely benefit from being independent of the athlete’s own employer, such as a recently introduced “red button” tool introduced by the international football players’ association (FIFPRO, 2020), as well as the integration of fraud-related issues in mental health support programs addressing the health of athletes (Reardon et al., 2019).

## Data Availability Statement

The original contributions presented in the study are included in the article/supplementary material, further inquiries can be directed to the corresponding author.

## Author Contributions

AH, CJ, and GK were all responsible of the overall design and research idea. AH wrote the first draft. All authors made substantial contributions to the editing of the paper and approved the final version.

## Conflict of Interest

Anders Håkansson holds a position as a professor at Lund University which is sponsored by the Swedish state-owned gambling operator AB Svenska Spel, and has research funding for projects (unrelated to the present one) from the research council of AB Svenska Spel and from the research council of Systembolaget, the Swedish alcohol monopoly, and from the Swedish Sport Federation. Funding organizations are unrelated to, and not involved in, the present paper. Caroline Jönsson is a board member of the FIFPRO and the Swedish Spelarföreningen (international and national soccer players’ unions). Göran Kenttä reports no conflicts of interest.

## Publisher’s Note

All claims expressed in this article are solely those of the authors and do not necessarily represent those of their affiliated organizations, or those of the publisher, the editors and the reviewers. Any product that may be evaluated in this article, or claim that may be made by its manufacturer, is not guaranteed or endorsed by the publisher.
